# *CDKN2A* copy number and p16 expression in malignant pleural mesothelioma in relation to asbestos exposure

**DOI:** 10.1186/s12885-019-5652-y

**Published:** 2019-05-28

**Authors:** Eeva Kettunen, Sauli Savukoski, Kaisa Salmenkivi, Tom Böhling, Esa Vanhala, Eeva Kuosma, Sisko Anttila, Henrik Wolff

**Affiliations:** 1 Research and Service Centre for Occupational Safety, Finnish Institute of Occupational Health, PO Box 40, FI-00032 Työterveyslaitos, Helsinki, Finland; 2 Department of Pathology, University of Helsinki, and HUSLAB, Helsinki University Hospital, 00029 HUS, Helsinki, Finland

**Keywords:** Malignant pleural mesothelioma, *CDKN2A*, p16, Tumor stroma, Asbestos exposure

## Abstract

**Background:**

Deletion of the *CDKN2A* locus is centrally involved in the development of several malignancies. In malignant pleural mesothelioma (MPM), it is one of the most frequently reported genomic alteration. MPM is strongly associated with a patients’ asbestos exposure. However, the status of *CDKN2A* and the expression of the corresponding protein, p16, in relation to MPM patient’s asbestos exposure is poorly known. Copy number alterations in 2p16, 9q33.1 and 19p13 have earlier been shown to accumulate in lung cancer in relation to asbestos exposure but their status in MPM is unclear.

**Methods:**

We studied DNA copy numbers for *CDKN2A* using fluorescence in situ hybridization (FISH) and p16 expression by immunohistochemistry (IHC) in 92 MPM patients, 75 of which with known asbestos exposure status. We also studied, in MPM, copy number alterations in 2p16, 9q33.1 and 19p13 by FISH.

**Results:**

We were unable to detect an association between p16 expression and pulmonary asbestos fiber count in MPM tumor cells. However, significantly more MPM patients with high pulmonary asbestos fiber count (> 1 million fibers per gram [f/g]) had stromal p16 immunoreactivity than MPM of patients with low exposure (≤ 0.5 million f/g) (51.4% vs 16.7%; *p* = 0.035, Chi-Square). We found that an abnormal copy number of *CDKN2A* in MPM tumor cells associated with a high pulmonary asbestos fiber count (*p* = 0.044, Fisher’s Exact test, two-tailed). In contrast to our earlier findings in asbestos associated lung cancer, DNA copy number changes in 2p16, 9q33 and 19p13 were not frequent in MPM although single cases with variable copy numbers on those regions were seen.

**Conclusions:**

We found two instances where the gene locus *CDKN2A* or its corresponding protein expression, is associated with high asbestos exposure levels. This suggests that there may be biological differences between the mesotheliomas with high pulmonary asbestos fiber count and those with low fiber count.

**Electronic supplementary material:**

The online version of this article (10.1186/s12885-019-5652-y) contains supplementary material, which is available to authorized users.

## Background

Asbestos fibers have been linked to mechanical and oxidative DNA damage through production of reactive oxidant species and fiber genotoxicity that can arise e.g. as genomic alterations [1–3]. Asbestos have been shown to have an important role in the etiology of some tumors such as malignant mesothelioma, lung cancer and laryngeal cancer [4, 5]. Alterations of *CDKN2A* locus and its corresponding protein expression are involved in numerous malignancies. In non-small cell lung cancer linked with asbestos exposure *CDKN2A* has been shown to be inactivated, mainly via deletions [6]. *CDKN2A/ARF* locus encodes tumor suppressor genes *p16*^*INK4A*^ and *p14*^*ARF*^ that interact with cyclin dependent kinase 4 (CDK4) and MDM2 proto-oncogene, respectively, and connect two important oncogenic pathways, RB and p53.

Malignant pleural mesothelioma (MPM) is a rare but deadly tumor type that is strongly associated with patients’ asbestos exposure [2]. Up to 80–90% of MPM in men is estimated to be associated with asbestos exposure [7]. In MPM, deletion of *CDKN2A* is the most frequently detected chromosomal change and the most common cause for p16 protein inactivation (reviewed in [8]). Hypermethylation of *CDKN2A* as a cause of loss of p16 expression in MPM has been reported in a minority of cases [9, 10]. The frequency of *CDKN2A* deletion in MPM have most often been shown to range from 61 to 88% in primary tumors, few studies, however, showing deletion only in one-fifth of cases [9, 11–20]. The *CDKN2A* deletions, detected by fluorescence in situ hybridization (FISH), have been exploited in differential diagnosis of MPM and benign mesothelial proliferations on effusions or biopsy material as well as in prognostication aims [13, 16, 20–24]. Expression of p16, however, cannot be used for these purposes [21]. Other genomic alterations (or their protein products) common in MPM such as in *BAP1* (BRCA1 associated protein 1), *MTAP* (methylthioadenosine phosphorylase) and *NF2* (neurofibromin 2) have also been studied to find out the most valuable marker combinations for differential diagnosis in MPM [25].

Only few studies - with a relatively limited number of patients - have evaluated the *CDKN2A*/p16 status in relation to asbestos exposure [9, 11, 13, 15]. These studies have shown either no significant association or a rather complex picture, partially the result was dependent on whether the exposure was assessed using occupational history or by asbestos fiber count. In this work we studied the relation between the patients’ pulmonary asbestos fiber counts determined by electron microscopy and the *CDKN2A*/p16 status in 92 MPM. Moreover, we report here for the first time p16 immunoreactivity in MPM in stromal cells and show a significant association between p16 positive stromal staining and asbestos exposure.

In MPM, the asbestos-related genomic changes reported mainly consist of copy number alterations such as deletions in 14q11.2-q21, 6q, 17p, and 22q and DNA methylation changes [26–28]. As we have earlier shown in lung cancer asbestos exposure-related accumulation of copy number alterations in 2p16, 9q33.1 and 19p13 [29, 30], we also studied the same loci in MPM. Contrary to lung cancer, MPM had normal mean copy number in 2p16, 9q33.1 and 19p13. Nevertheless, in these loci the individuals in high asbestos exposure group showed wider ranges of DNA copy number than the low exposure group.

## Methods

### Malignant pleural mesothelioma tissue samples

Study material consisted of 92 formalin-fixed paraffin-embedded (FFPE) MPM specimens from Caucasian patients. Demographic data of the study subjects are shown in Table 1. Pathology samples used for tissue microarrays (for FISH or IHC) have originally been collected in Central Hospitals in Finland as diagnostic specimens. Autopsy specimens for pulmonary fiber count measurements have originally been collected for forensic purposes. Guidelines for this sampling of autopsy specimens require that they represent an area without tumor or fibrosis, at an intermediate distance from bronchus and pleura. Before electron microscopy analysis the samples were also visually inspected at the pathology laboratory of the Finnish Institute of Occupational Health. MPMs displayed either epithelioid, sarcomatoid or biphasic histology. Diagnoses of patients were confirmed by expert pathologists and in borderline cases by consensus [31, 32].

**Table 1:** Demographic data of the study subjects in MPM microarrays

	Malignant pleural mesothelioma *n* = 92			Quality controls^b^
	Asbestos fiber count^a^ ≥ 1.0 × 10^6^ f/g	Asbestos fiber count^a^ 0–0.5 × 10^6^ f/g	Fiber count not available	
	*n* = 53	*n* = 22	*n* = 17	*n* = 7
Gender, male	50 (94%)	16 (73%)	8 (47%)	2 (29%)
Age, mean ± SD, y	63.7 ± 8.7	68.1 ± 10.2	61.5 ± 8.3	56.9 ± 9.4
Pulmonary fiber count^a^, median (range), million f/g	8.9 (1.1–1000)	0.2 (0–0.5)	NA	0.8 (0.4–16.0)^c^
Mesothelioma histologic type				
Epithelioid	43 (81%)	11 (50%)	9 (53%)	–
Biphasic	4 (8%)	6 (27%)	3 (18%)	–
Sarcomatoid	6 (11%)	5 (23%)	5 (29%)	–

^a^Pulmonary asbestos fiber counts by scanning electron microscopy (SEM) or transmission electron microscopy (TEM): million fibres per gram dry lung (detection limit ~ < 0.1 million f/g); ^b^Methodologic quality controls had either serous ovarian carcinoma, serous ovarian or peritoneal carcinoma, adenocarcinoma of the lung or pleomorphic liposarcoma; ^c^Fiber counts were known for three cases only, thus controls were not used in asbestos-related analysis

Pulmonary asbestos fiber counts, using fiber length of > 1 μm, for 75 of the MPM patients could be analyzed using electron microscopy with energy dispersive spectrometry [33] (Table 1). In an international recommendation concerning the attribution of asbestos-related diseases to asbestos exposure, a pulmonary fiber count > 1 million fibers per gram dry weight of lung tissue (f/g) is recommended to identify individuals with a high probability of asbestos exposure at work [34]. In this article a pulmonary fiber count > 1 million f/g is referred to as a high exposure and ≥ 5 million f/g to as very high exposure. A pulmonary fiber count ≤0.5 million f/g is referred to as a low asbestos exposure. To heighten the contrast between high and low exposure, eight specimens from MPM patients having pulmonary fiber count between 0.5 and 1 million f/g were excluded (data not shown in Table 1).

### Tissue microarrays

Tissue microarray (TMA) blocks of MPM specimens were built using Beecher instrument. Four cores of 0.1 cm in diameter were obtained for each case. If the original sample available was very small, four cores of 0.06 cm in diameter or minimum two cores of 0.1 cm in diameter were obtained. TMA blocks included as methodologic quality control specimens some non-tumorous pleura/lung and seven tumors other than malignant mesothelioma (MM) (serous ovarian or serous peritoneal carcinoma from five females, lung adenocarcinoma and pleomorphic liposarcoma from two males). Twenty-six serous ovarian carcinomas in TMA from an earlier study were used as positive p16 expression controls [35].

Routine H&E staining of sections were studied to evaluate the tumors. Because some sample cores were absent in TMA slides, somewhat different number of tumors produced results in FISH and IHC study, the numbers of tumors being shown in each table.

### Immunohistochemistry (IHC)

For IHC, TMA sections of 2.5 μm were treated for antigen retrieval in Lab Vision™ PT-module (Thermo Fisher Scientific Inc., Fremont, USA) using citrate buffer, pH 6.0 for 10 min (Thermo Fisher Scientific Inc.). Immunostaining was done in Lab Vision™ Autostainer (Thermo Fisher Scientific Inc.) using a 1:200 dilution of mouse monoclonal antibody for p16 (JC8) (Santa Cruz Biotechnology, Inc., Santa Cruz, California, USA) for 60 min at RT. For detection we used BrightVision plus Poly-HRP-Anti Ms./Rb/Rt IgG (ImmunoLogic, Duiven, Netherlands) with chromogen AEC (Thermo Fisher Scientific Inc.) and Mayer haematoxylin as a background stain. Our p16 staining was further validated by comparing it to that of an accredited medical pathology laboratory (HUSLAB, Helsinki, Finland) p16 staining using CINtec® p16 Histology clone E6H4™ (mtm laboratories AG, Roche Diagnostics GmbH, Mannheim, Germany) and DAP. These stainings were made from whole tissue sections of three cases with distinctive staining patterns. The positive controls of twenty-six serous ovarian carcinomas were stained for p16 using CINtec® p16 Histology clone E6H4™. Immunostained preparations were reviewed by HW without prior knowledge of the exposure status using an Olympus BH2 (Olympus Europa SE & Co. KG, Hamburg), and photographed using Leica microscope & camera system with Leica Application Suite V4.10 [Leica Microsystems (Switzerland) Limited]. Scoring for positivity was as follows: 0–1% = negative, 2–10% = +, 11–50% = ++, and 51–100% = +++. For some statistical analysis, the result was dichotomized into negative and positive (+/++/+++) immunoreactivity. Minimum of two TMA cores per sample were required and the highest results among the cores were recorded. Tumor cells and stromal cells were evaluated separately. For sarcomatoid MM, stromal cells were not evaluated. Serous ovarian or serous peritoneal carcinoma from five females, lung adenocarcinoma and pleomorphic liposarcoma from two males served as quality controls in MPM TMAs. Twenty-six serous ovarian carcinomas in a separate TMA served as positive control cases for p16 expression.

### Fluorescence in situ hybridization (FISH)

We studied DNA copy numbers in TMAs for *CDKN2A* at 9p21 and centromere 9 (CEP9) simultaneously in each cell, using a dual color probe mix of centromeric probe labeled with Spectrum (Sp.) Green and *CDKN2A* locus specific probe with Sp. Orange (Vysis Inc./ Abbott Molecular Inc., Downers Grove, IL, USA). *CDKN2A* copy number was considered as abnormal when > 20% of the cells had lost both *CDKN2A* signals but showed at least one CEP9 (i.e. homozygous deletion, HD), or > 20% of the cells had only one *CDKN2A* signal or at least lower signal number than that of CEP9 (hemizygous deletion), or ≥ 50% of the cells had one *CDKN2A* and one CEP9 signal (monosomy).

Locus specific DNA copy numbers in 2p16, 9q33.1 and 19p13 were studied in TMAs using bacterial artificial chromosomes (BAC) as described earlier [36]. Copy numbers of chromosomes 2, 9, 10 and 15 centromeres were studied using a FISHBright CEP9 probe (Qbiogene, Illkirch, France, and Kreatech Biotechnology BV, Amsterdam, The Netherlands), CEP2 alpha satellite DNA probe, Sp. Green-labeled centromere 9 and centromere 10 probes, and Sp. Orange-labeled centromere 15 probe (Vysis Inc./Abbott Molecular, Inc.). Hybridizations were performed according to the manufacturers’ instructions. 2p16, 9q33.1, and 19p13 were compared with centromere copy numbers as described earlier [29]. Centromeric probe for chromosome 19 is unavailable, thus 19p13 signals were divided by an average of centromeric signals of #2, #9, #10, and #15.

FISH preparations were analysed using a Zeiss AxioImager.Z1 fluorescence microscope (Zeiss, Jena, Germany), without prior knowledge of the exposure status or clinico-pathological data. Efficiency of hybridization was evaluated with lymphocytes serving as internal controls. FISH signals were scored in 30 to 200 non-overlapping interphase nuclei.

### Statistical methods

Chi-Square test and Fisher’s Exact test were used to test the differences between asbestos fiber exposure groups (very high, high, low). To measure the degree of agreement with *CDKN2A* abnormality and p16 staining we calculated Cohen’s Kappa (κ) Coefficient.

## Results

MPM tumors from 92 patients having either low (≤0.5 × 10^6^ f/g), high (≥1.0 × 10^6^ f/g), including very high (≥ 5.0 × 10^6^ f/g), or unknown pulmonary asbestos fiber counts (unknown exposure) were studied for p16 immunoreactivity and *CDKN2A* copy number. Furthermore, DNA copy numbers were evaluated in genomic regions 2p16, 9q33.1, and 19p13 that earlier in lung cancer have been found with accumulating changes that associated with asbestos exposure [29]. Some sample cores being absent in some slides, somewhat different number of tumors produced results in FISH and IHC study. The numbers of tumors have been shown in each table. Result for p16 was available for 69 MPM and for both p16 and *CDKN2A* copy number were available for 62 MPM. For 65 MPM, DNA copy numbers was obtained in one to three of the 2p16, 9q33.1, and 19p13 loci, as proportional to centromere.

### Protein expression of p16 in MPM

**Fig. 1:**
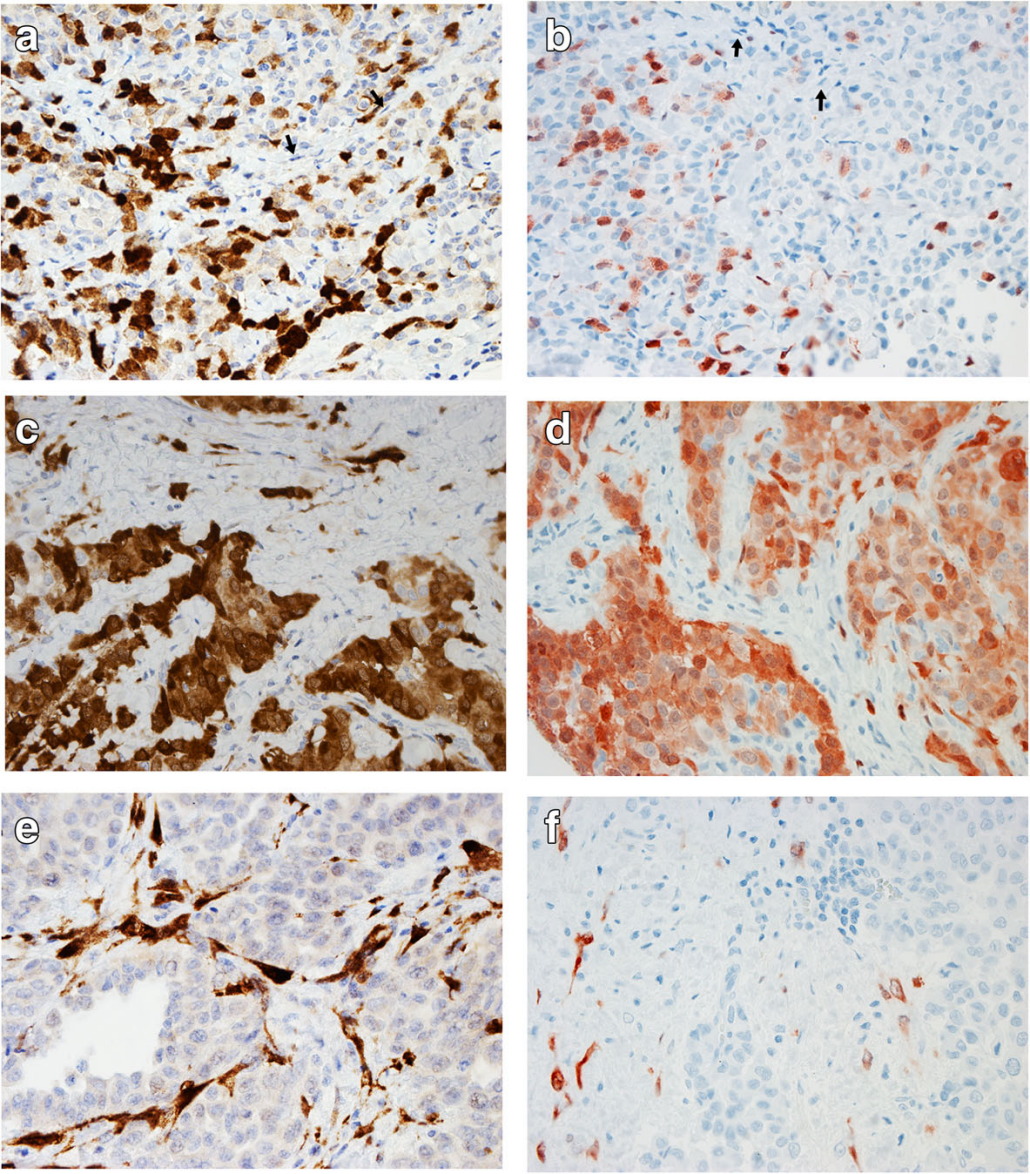
p16 staining in epithelioid malignant pleural mesothelioma (MPM-E). Both the whole section (**a**, **c**, and **e**; using CINtec® p16 Histology clone E6H4™ with DAP as chromogen) and tissue array (TMA) cylinder samples (**b**, **d**, and **f**; using mouse monoclonal antibody for p16 [JC8] with AEC as chromogen) are shown. All microscopic illustrations have magnification × 40. Panels **a**) and **b**) show p16 staining in the case E-161 who had pulmonary asbestos fiber count of 0.4 × 10^6^ fibers / gram dry weight (f/g). Tumor cells showed positivity of +++ (**a**) to ++ (**b**) while stromal cells (arrows) were negative for p16. Panels **c**) and **d**) show p16 staining in the case E-143 who had pulmonary asbestos fiber count of 8.9 × 10^6^ f/g. Tumor cells showed strong positivity and stromal cells had positivity of ++ (**c**) to + (**d**). Panels **e**) and **f**) show p16 staining in the case E-250 who had pulmonary asbestos fiber count of 13.0 × 10^6^ f/g. Tumor cells were negative for p16 whereas stromal cells showed positivity of +++ (**e**) to ++ (**f**)

Protein expression of p16 was evaluated by IHC in tumor cells and stromal cells in TMA core preparations (Table 2). Three of the MPMs were also studied as an entire tissue section preparation and showed similar staining pattern as in TMA, supporting our approach (Fig. 1). p16 staining status did not associate with gender, age, or mesothelioma histology, neither in MPM tumor nor stromal cells as shown in Table 2. By contrast, significantly more MPM patients with high asbestos exposure had p16 positive stromal staining (*p* = 0.035, Chi-Square) than had MPM of patients with low exposure (51.4% vs 16.7%). Positivity of p16 in stromal cells was even more predominant among the very highly exposed when compared with MPM with low exposure (*p* = 0.018, Chi-Square) (Table 2). In MPM tumor cells, an association between p16 expression and the asbestos fiber count was not shown.

**Table 2:** Association of gender, age, histologic type, and exposure with p16 expression and *CDKN2A* copies in MPM

				All	Gender		Age^a^		Mesothelioma (MPM) histologic types			Asbestos exposure*,* all MPM histologic types^b^		Very high vs. low exposure, all MPM histologic types^b^		Asbestos exposure, epithelioid histology	
					Male	Female	<Median 62.5y	≥Median 62.5y	Epith.	Biphasic	Sarcom.	High^c^	Low^d^	Very high^e^	Low^d^	High^c^	Low^d^
			n^f^	92	74	18	46	46	63	13	16	53	22	33	22	43	11
p16 staining by IHC	MPM tumor cells	neg	n (%)	50 (73)	42 (75)	8 (62)	24 (71)	26 (74)	37 (73)	7 (100)	6 (55)	34 (81)	10 (67)	21 (81)	10 (67)	30 (81)	4 (50)
		pos	n (%)	19 (27)	14 (25)	5 (38)	10 (29)	9 (26)	14 (27)	0 (0)	5 (45)	8 (19)	5 (33)	5 (19)	5 (33)	7 (19)	4 (50)
	*p*				0.491^g^		0.729^h^		0.107^g^			0.294^g^		0.453^g^		0.085^g^	
	MPM stromal cells	neg	n (%)	30 (53)	23 (51)	7 (58)	15 (52)	15 (54)	25 (51)	5 (63)	–	18 (49)	10 (83)	10 (42)	10 (83)	18 (51)	6 (75)
		pos	n (%)	27 (47)	22 (49)	5 (42)	14 (48)	13 (46)	24 (49)	3 (37)	–	19 (51)	2 (17)	14 (58)	2 (17)	17 (49)	2 (25)
	*p*				0.655^h^		0.888^h^		0.709^g^			0.035*^h^		0.018*^h^		0.270^g^	
*CDKN2A* copy number by FISH	MPM tumor cells	abnorm^i^	n (%)	53 (84)	45 (90)	8 (62)	27 (90)	26 (79)	40 (89)	6 (75)	7 (70)	34 (94)	10 (71)	21 (96)	10 (71)	31 (97)	4 (57)
		norm	n (%)	10 (16)	5 (10)	5 (38)	3 (10)	7 (21)	5 (11)	2 (25)	3 (30)	2 (6)	4 (29)	1 (4)	4 (29)	1 (3)	3 (43)
	*p*				0.025*^g^		0.308^g^		0.170^g^			0.044*^g^		0.064^g^		0.014*^g^	

Association of gender, age, histologic type, and exposure with p16 expression and *CDKN2A* copy number status in malignant pleural mesothelioma (MPM)

^a^Age at time of asbestos-analysis (i.e. surgery or autopsy); ^b^For stromal p16 staining analysis, sarcomatoid cases were excluded; ^c^Pulmonary asbestos fiber count ≥1.0 × 10^6^ fibres per gram dry lung; ^d^Pulmonary asbestos fiber count 0–0.5 × 10^6^ fibres per gram dry lung; ^e^Pulmonary asbestos fiber count ≥5.0 × 10^6^ fibres per gram dry lung; ^f^Total number of studied cases; ^g^Fisher’s Exact test (two-tailed). Tested for negative/abnormal and positive/normal results between the sub-categories; ^h^Chi-Square. Tested for negative/abnormal and positive/normal results between the sub-categories; ^i^*CDKN2A* copy number was considered as abnormal if homozygous deletion was shown in > 20% of the cells or hemizygosity in > 20% of the cells, or monosomy of chromosome 9 in ≥50%;

*MPM* malignant pleural mesothelioma, *IHC* immunohistochemistry, *FISH* fluorescence in situ hybridization, *y* years, *P*-values ≤0.05 with *

We also studied the relation of p16 staining between stromal and tumor cells in each individual sample (Table 3). Fifteen (39.5%) patients with high asbestos count had some staining in MPM stroma but not in tumor cells whereas only one (8.3%) patient in low exposure group stained this way (*p* = 0.048, Fisher exact test, two-tailed) (Table 3). One of the positive controls stained in this way.

**Table 3:** MPM and controls analyzed in relation with tumor and stromal mark in p16 IHC staining

	Malignant pleural mesothelioma		Positive controls	Quality controls
p16 staining by tumor – stroma relation	High exposure^a^ *n* = 47^b,c^	Low exposure^d^ n = 17^e^	*n* = 26^f^	*n* = 7
Tumor neg, Stroma neg				
n (%)	17 (45)	7 (59)	1 (4%)	2 (~ 33%) ^h^
*p*^g^	0.774			
Tumor pos, Stroma neg				
n (%)	2 (5)	3 (25)	4 (15%)	2 (~ 33%)^i^
*p*^g^	0.112			
Tumor neg, Stroma pos				
n (%)	15 (40)	1 (8)	1 (4%)	0 (0%)
*p*^g^	0.048*			
Tumor pos, Stroma pos				
n (%)	4 (10)	1 (8)	20 (77%)	2 (~ 33%)^j^
*p*^g^	1.000			

Epithelioid and biphasic malignant pleural mesothelioma and controls analyzed in relation with tumor and stromal mark in p16 immunohistochemical (IHC) staining. ^a^Pulmonary asbestos fiber count ≥1.0 × 10^6^ fibres per gram dry lung (f/g); ^b^For two cases, tumor stained positive but stroma could not be analyzed; ^c^For eight cases IHC staining was not available; ^d^Pulmonary asbestos fiber count 0–0.5 × 10^6^ f/g; ^e^For five cases IHC staining was not available; ^f^Positive controls were serous ovarian carcinomas; ^g^Fisher’s Exact Test, two-tailed; ^h^Adenocarcinoma of the lung and pleomorphic liposarcoma; ^i^Serous ovarian carcinoma and serous ovarian or peritoneal carcinoma, ^j^Serous ovarian carcinoma and serous peritoneal carcinoma; for one serous ovarian or peritoneal carcinoma IHC staining was not available.

### DNA copy number of *CDKN2A* in relation to centromere 9 (CEP9) in MPM

**Fig. 2:**
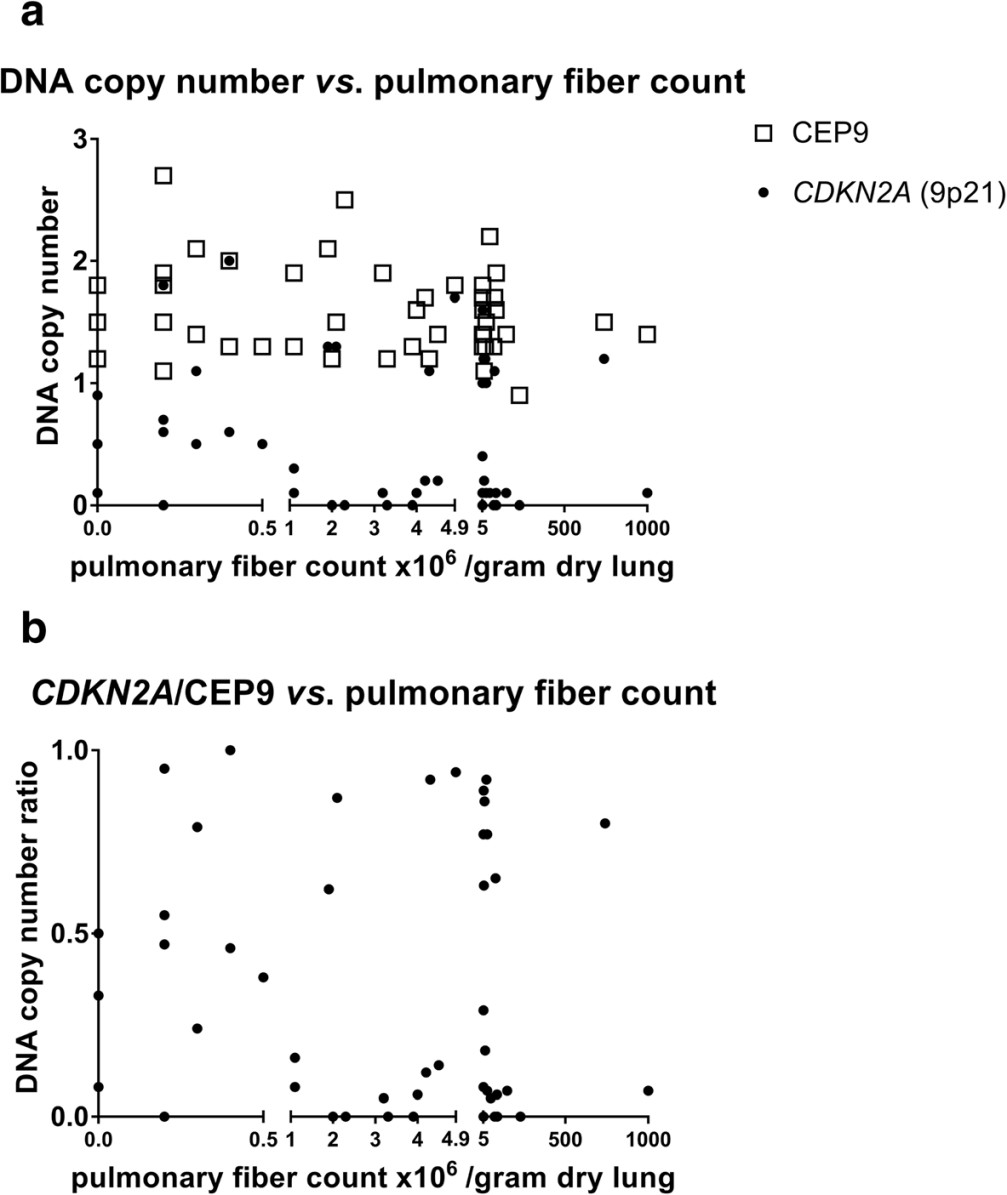
Scatter plots showing the relation of the pulmonary asbestos fiber count in each individual suffering from malignant pleural mesothelioma and **a**) the mean DNA copy numbers of *CDKN2A* at 9p21 and the centromeric locus of chromosome 9 (CEN9) or **b**) the ratio of the mean DNA copy numbers of *CDKN2A* and CEN9 in the tumors of the patients. The x-axis scale is discontinuous to provide dynamic range from the low (≤0.5 × 10^6^ f/g) to high (≥1.0 × 10^6^ f/g) and to very high fiber counts (≥5.0 × 10^6^ f/g)

Using FISH, copy numbers for *CDKN2A* in 9p21 and CEP9 could be scored in 63 MPM. Homozygous deletion (HD) was the most common abnormality shown in 65% of the cases. Hemizygosity or monosomy of the chromosome 9 were both shown in 9.5% whereas 16% of the MPM had normal copy number. Mean signal counts of *CDKN2A* and CEP9 were 0.6 and 1.54 in MPM patients with high asbestos exposure whereas in low exposure patients *CDKN2A* and CEP9 counts were 0.8 and 1.7 (Additional file 1). The proportions of copy number statuses in relation to various clinico-pathological features are shown in Table 2. Abnormal DNA copy number of *CDKN2A* in MPM tumor cells associated with patients’ high pulmonary asbestos fiber count (*p* = 0.044, Fisher’s Exact test, two-tailed) (Table 2, Fig. 2). This association was strongest among epithelioid MPM (*p* = 0.014, Fisher’s Exact test, two-tailed) (Table 2). In Additional files 2 and 4, examples of FISH preparations in MPM with different amount of pulmonary asbestos fibers are shown. The *CDKN2A* copy number did not relate with age of the patient or MPM histologic type. In contrast, there was an association with gender (*p* = 0.025, Fisher’s Exact test, two-tailed) (Table 2) which reflected the higher proportion of subjects with high asbestos exposure among males (76%) in comparison to females (33%) (Table 1).

When the relation of *CDKN2A* abnormality and p16 staining status was observed in individual MPM tumors, low exposure group tumors with abnormal *CDKN2A* always were negative for p16 staining and vice versa, yet the case numbers were small (Kappa coefficient 1.000) (Additional file 3). Other study groups did not show similar pattern of relation between *CDKN2A* copy number and p16 staining. Additional file 4 (g, h, j) shows a sarcomatoid MPM (exposure unknown) with *CDKN2A* homozygosity in 74% of the tumor cells and normal copy number of *CDKN2A* and CEP9 in adjacent hepatic cells, both being negative for p16.

### DNA copy numbers in 2p16, 9q33.1, 19p13, and chromosome 2, 9, 10, and 15 centromeres

For 65 MPM, FISH result was obtained in one to three of the 2p16, 9q33.1, and 19p13 loci, mean count of each chromosome centromere serving as a baseline. Mean signal counts of the loci and centromeres #2, #9, #10 and #15 suggested that copy numbers in 2p16, 9q33.1, and 19p13 and centromeres of chromosomes 2, 9, 10, and 15 in majority of MPM were diploid or close to diploid (Additional file 1). Nevertheless, there were some difference in ranges of the signal counts between the high exposure and low exposure groups as well as in the ranges of mean signal ratios (Additional file 1), the high exposure group having wider ranges of chromosomal abnormality.

## Discussion

Asbestos exposure causes mechanical and oxidative DNA damage and is linked to genomic alterations [1, 2]. In mouse studies, MM formation through asbestos exposure has shown a significant role of alterations in *Cdkn2a/Arf* [37] and deletion of *CDKN2A* is the most frequently detected chromosomal change in human MPM [8]. Fiber-induced *CDKN2A* disruption has also been observed in a study in which mesothelioma was induced in mice by instillation of either asbestos fibers or long-fiber carbon nanotube (CNTs) into the pleural cavities [38].

We studied the expression of p16 encoded by *CDKN2A* in MPM from 92 patients with different asbestos-burdens. In the MPM tumor cells, we did not detect an association between p16 expression and asbestos fiber exposure as determined by asbestos fiber count. However, significantly more MPM patients with high pulmonary asbestos fiber count had stromal p16 immunoreactivity than MPM patients with low exposure. The p16 positive stromal staining was often present concurrently with negative p16 staining in tumor cells. To our knowledge earlier studies have not reported p16 immunoreactivity of stromal cells in MPM.

In gynecological malignancies, p16 overexpression in tumor cells is widely used diagnostic marker because it associates with HPV infection indicating a higher risk for cancer. Recently, increased stromal expression of p16, showing gradual increase with the level of malignancy, has been reported in some tumors such as malignant ovarian carcinoma and endometrial carcinoma [39, 40]. However, the significance of stromal p16 immunoreactivity status is not clear.

It has been shown that increased expression of p16 could induce senescence without a senescence-associated secretory phenotype and limit the accumulation of DNA damage [41]. Our result of stromal p16 expression in highly asbestos exposed patients’ MPM may mark a response to cell activation caused by fibers but further studies are needed. Mouse studies have produced experimental data about some molecular changes related to fiber exposure as described above [37, 38] whereas in human MPM asbestos-related changes reported are mainly copy number alterations and DNA methylation changes [26–28]. There are some reports suggesting that mesotheliomas associated with asbestos exposure have a poorer prognosis than those not associated with the exposure [42–45].

The most frequently reported chromosomal alteration in MPM (61% ─ 88%) is deletion of material in 9p21, i.e. primarily *CDKN2A* encoding tumor suppressors p16INK4A and p14ARF [9, 11–13, 15, 16, 18–20]. Accordingly, a majority (84.1%) of our MPM cases showed a *CDKN2A* copy number abnormality, either HD or hemizygous deletion or monosomy. The LSI *CDKN2A* probe we utilized covers also CDKN2B and MTAP encoding sequences in 9p21. In line with our result, a comprehensive cytogenetic study of 17 MM cell lines has shown that the above-mentioned genes in the heart of the region showed HD in 82–100% of the cell lines [46]. Moreover, there were numerous other genes in 9p21 showing HD or allelic loss in 12–65% of those MM cell lines and other additional complex chromosome 9 rearrangements being identified by combined chromosomal techniques [46].

In our study, asbestos exposure of the MPM patients has been evaluated using pulmonary asbestos fiber counts measured with electron microscopy. We showed that abnormal copy number of *CDKN2A* in MPM tumor cells associated with high pulmonary asbestos fiber count but not with age or MPM histologic type. An earlier study of Hirao et al. showed a somewhat complex relation between asbestos exposure and *p16* changes. In their study, *p16* alterations (deletion or methylation) were more common in cases with lower pulmonary asbestos (fiber counts by a method different than ours) although *p16* deletions were more common in heavy exposed group. In that study, part of asbestos exposure of the subjects had been evaluated by medical or occupational history [15]. In other earlier studies, exposure data obtained from work history evaluation, *CDKN2A* deletion and asbestos exposure did not associate with each other [9, 11, 13].

We found here centromere 9 (CEP9) to be diploid when using the single probe. With the dual probe mix including probes for CEP9 and *CDKN2A*, somewhat lowered mean CEP9 signals were counted. This could be due to the different probe used in these two analysis types and heterogeneous specimens of genetically disturbed cancer cells with variable changes. When establishing cut-off values for *CDKN2A* FISH protocol, Chung et al. have shown a pattern of ‘one *CDKN2A* signal/one CEP9 signal’ in 6–34% of nuclei in reactive mesothelial proliferation and further in 5% of nuclei in MPM [13]. In MM cell lines, chromosome 9 fragments rather than whole 9 have been shown affected by HD, chromosomal aberrations being though various and very complex in MM [46]. Our CEP9 results are in line with those reports.

This is the first report indicating a correlation between the *CDKN2A* deletion status and the p16 protein levels in MPM patients having low asbestos exposure. Earlier, somewhat discrepant results about the relationship and correlation of the *CDKN2A* deletion status and the p16 levels in MPM has been reported [9, 14, 20, 21]. Immunoreactivity for the proteins p14, p15, p16 and MTAP, all encoded from DNA in 9p21, has been studied and compared with FISH result of 9p21. MTAP protein expression has been reported to correlate best with HD of *CDKN2A* [47]. There are also other mechanisms such as DNA methylation that can influence protein expression [10]. In our study, the *CDKN2A* probe in 9p21 we used covers also *MTAP* and *CDKN2B* whereas the antibody in immunohistochemistry recognized specifically CDKN2A/p16. This could cause some lack of correlation in the high exposure MPM and controls. Moreover, *CDKN2A* copy number in majority of MPM is abnormal and thus there are relatively small numbers of cases with normal *CDKN2A* copy number available for a statistical comparison. Nevertheless, the correlation between the *CDKN2A* deletion status and p16 protein levels was shown here for the first time, interestingly only in the low exposure group MPM. The significance of this finding is unclear, but it is in line with the other findings in this paper that suggest differences in various aspects of *CDKN2A*/p16 depending on the level of asbestos exposure.

Our studies here on locus specific DNA copy numbers and on the centromeres of chromosomes 2, 9, 10 and 15 suggested that asbestos exposure related accumulation of alterations in 2p16, 9q33 and 19p13 that we earlier have showed in lung cancer [29, 30] was not comparable in MPM. Yet we found out that the ranges of signal count in 19p13 and 9q33 in MPM were wider with high exposure group than with low exposure group despite the normal mean signal count. Abnormal signals, even if being infrequent may suggest that in asbestos-exposed patients these genomic regions gained disintegration due to exposure-induced DNA damage, reflected as increased locus copy number variation in single MPM individuals.

Interestingly, loss in 19p13.2 coinciding with the sequence that is recognized by our 19p13 probe has earlier been reported in 55% of MPM but without an association to asbestos exposure probably due to the small number of non-exposed patients in that study [48]. Although 2p changes were not reported using comparative genomic hybridization in primary MPM tumors [49], another study on MPM cell lines have shown gains in 2p16.2-p12, 2p11.2-q11.2, and 19p13.13, depending on the cell passage [50].

## Conclusions

In conclusion, we report here for the first time that p16 immunoreactivity in stromal cells of MPM had significant relation with the patients’ high asbestos exposure, suggesting that the biology of MPM in highly exposed individuals differed from that of mesotheliomas in subjects with low exposure. Additionally, abnormal copy number of *CDKN2A* in MPM tumor cells associated with high pulmonary asbestos fiber count, again suggesting a biological difference between mesotheliomas depending on the degree of asbestos exposure. In contrast to the situation in lung cancer, asbestos exposure associated molecular changes in 2p16, 9q33 and 19p13 were not frequent in MPM though single cases with variable DNA copy numbers on those regions were seen.

## Additional files

Additional file 1: A table - Mean signal counts of fluorescent locus specific probes and centromeric CEP probes in different study groups of malignant pleural mesothelioma in FISH analysis. (PDF 703 kb)

Additional file 2: Figure legend for Additional file 4. (PDF 1005 kb)

Additional file 3: A table - Relation of the *CDKN2A* copy number and p16 staining status in tumor cells of malignant pleural mesothelioma patients in general and with different asbestos-burden and in quality controls. (PDF 762 kb)

Additional file 4: A pdf file of images of FISH and IHC preparations of malignant pleural mesothelioma (MPM). (PDF 2607 kb)

## Abbreviations

BAC: Bacterial artificial chromosome
BAP1: BRCA1 associated protein 1
CDK4: Cyclin dependent kinase 4
CEP: Centromere
CNT: Carbon nanotube
f/g: Fibers per gram
FFPE: Formalin-fixed paraffin-embedded
FISH: Fluorescence in situ hybridization
HD: Homozygous deletion
IHC: Immunohistochemistry
MM: Malignant mesothelioma
MPM: Malignant pleural mesothelioma
MTAP: Methylthioadenosine phosphorylase
NF2: Neurofibromin 2
Sp: Spectrum
TMA: Tissue microarray
