# Supplementary material for: CDKN2A copy number and p16 expression in malignant pleural mesothelioma in relation to asbestos exposure
Source: BMC Cancer. 2019 May 28;19:507. doi: 10.1186/s12885-019-5652-y (PMC6537412; doi:10.1186/s12885-019-5652-y)
Supplement: Supplementary file 1 — A table - Mean signal counts of fluorescent locus specific probes and centromeric CEP probes in different study groups of malignant pleural mesothelioma in FISH analysis. (PDF 703 kb) [file 12885_2019_5652_MOESM1_ESM.pdf]

Additional Files_1
"*CDKN2A* copy number and p16 expression in malignant pleural mesothelioma in relation to asbestos exposure"
by Kettunen E, Savukoski S, Salmenkivi K, Böhling T, Vanhala E, Kuosma E, Anttila S, Wolff H.
Eeva Kettunen, PhD; Finnish Institute of Occupational Health, Finland; Mar 19, 2019, eeva.kettunen@ttl.fi

Additional File 1. Mean signal counts of fluorescent locus specific probes and centromeric CEP probes (and ratios in the footnote) in different study groups of malignant pleural mesothelioma in fluorescence in situ hybridization (FISH) analysis.

	Malignant pleural mesothelioma			
DNA locus	High exposure[a]	Low exposure[b]	Unknown exposure	Quality controls[c]
	**Mean signal count (range)**<br>**n**			
CDKN2A (9p21)	0.6 (0-2.2)<br>37	0.8 (0-2.0)<br>14	0.8 (0-1.8)<br>13	0.84 (0.1-1.7)<br>7
CEP9 (in dual probe[d])	1.54 (0.9-2.5)<br>37	1.7 (1.1-2.7)<br>14	1.42 (1.1-2.0)<br>13	1.46 (1.1-1.8)<br>7
2p16	1.9 (1.1-2.2)[e]<br>27	1.9 (1.2-2.2)[f]<br>15	1.9 (1.4-2.2)<br>12	-
9q33	1.8 (1.1-2.0)[g]<br>20	1.9 (1.6-2.0)[h]<br>6	2.0<br>1	-
19p13	2.0 (1.2-3.1)[i]<br>15	2.0 (1.8-2.1)[j]<br>10	2.1 (1.4-2.8)<br>6	-
CEP2	2.0 (1.5-2.5)<br>28	2.0 (1.7-2.1)<br>15	2.0 (1.7-2.3)<br>13	-
CEP9	1.8 (1.1-2.0)<br>24	2.0 (1.9-2.0)<br>8	2.0 (1.5-2.2)<br>6	-
CEP10	1.9 (1.6-2.1)<br>19	1.9 (1.5-2.0)<br>8	1.9 (1.9-1.9)<br>4	-
CEP15	2.0 (1.3-2.9)<br>20	2.0 (1.9-2.1)<br>11	1.9 (1.6-2.3)<br>7	-

[a]Patients' pulmonary asbestos fiber count ≥1.0 x10^6 fibres per gram dry lung (f/g); [b]Patients' pulmonary asbestos fiber count 0-0.5 x10^6 f/g; [c]Quality controls had either serous ovarian or serous peritoneal carcinoma, adenocarcinoma of the lung or pleomorphic liposarcoma; [d]A dual color probe mix of centromeric probe labeled with Spectrum (Sp.) Green and *CDKN2A* locus specific probe with Sp. Orange (Vysis Inc./ Abbott Molecular Inc., Downers Grove, IL, USA); [e]Mean signal ratio (range) in 2p16/CEP2 was 1.0 (0.7-1.3) in high exposure group and [f]1.0 (0.6-1.1) in low exposure group; [g]Mean signal ratio (range) in 9q33/CEP9 was 1.0 (0.6-1.6) in high exposure group and [h]1.0 (0.8-1.1) in low exposure group; [i]Mean signal ratio (range) in 19p13/mean CEPs was 1.1 (0.7-1.6) in high exposure group and [j]1.0 (0.9-1.2) in low exposure group
