# Supplementary material for: CDKN2A copy number and p16 expression in malignant pleural mesothelioma in relation to asbestos exposure
Source: BMC Cancer. 2019 May 28;19:507. doi: 10.1186/s12885-019-5652-y (PMC6537412; doi:10.1186/s12885-019-5652-y)
Supplement: Supplementary file 2 — Figure legend for Additional file 4. (PDF 1005 kb) [file 12885_2019_5652_MOESM2_ESM.pdf]

Additional File_2

"CDKN2A copy number and p16 expression in malignant pleural mesothelioma in relation to asbestos exposure"

by Kettunen E, Savukoski S, Salmenkivi K, Böhling T, Vanhala E, Kuosma E, Anttila S, Wolff H.
Eeva Kettunen, PhD; Finnish Institute of Occupational Health, Finland; Mar 19, 2019,
eeva.kettunen@ttl.fi

Additional File 2. Figure legend for Additional File 4.(Figure attached as **Additional File 4**) Fluorescence in situ hybridisation (FISH) preparations of malignant pleural mesothelioma (MPM). A dual color probe mix (Vysis Inc./ Abbott Molecular Inc.) showed green centromeric #9 signals and orange *CDKN2A* locus specific signals. FISH illustrations have magnification x40. Pulmonary asbestos fiber counts are presented for each case whether available or not (NA). Counts are recorded as million (m) fibres per gram of dry lung (f/g), measured with electron microscopy.

a) In an epithelioid MPM, case E-250, *CDKN2A* was hemizygously deleted in 21% of the tumor cells. [In immunohistochemistry (IHC) of the case 250, tumor cells were negative for p16 whereas stromal cells stained strongly, shown in Fig.1e and f]

b) In a sarcomatoid MPM, case S-4, *CDKN2A* was hemizygously deleted in 49% of the tumor cells: the case displayed nuclei with amplified CEP9 with one CDKN2A signal (arrows) [IHC of the case S-4, tumor cells showed negative staining for p16, stromal cells were not evaluated in sarcomatoid cases; not illustrated here]

c) In an epithelioid MPM, case E-31, *CDKN2A* was homozygously deleted in 99% of the tumor cells [IHC showed negative p16 staining for both tumor and stromal cells of the case E-31; not illustrated here]

d) In an epithelioid MPM, case E-272, *CDKN2A* was deleted homozygously in 28% and hemizygously in 12% (arrow in the lower corner) of the tumor cells. Furthermore, monosomy of chromosome 9 was seen in 41% of the tumor cells (upper arrow) [in IHC of the case E-272, tumor cells were negative for p16 and stromal cells stained +; not illustrated here]

e) In a mixed MPM, case M-234, *CDKN2A* showed homozygous deletion in 91% of the tumor cells, [in IHC of the case M-234, tumor cells were negative for p16 whereas stromal cells stained ++; not illustrated here]

f) In a mixed MPM, case M-141, *CDKN2A* was deleted homozygously in 4% and hemizygously (arrows) in 16% of the tumor cells, [in IHC of the case M-141, tumor cells were negative for p16 whereas stromal cells stained ++; not illustrated here]

g) In a sarcomatoid MPM, case S-12, *CDKN2A* was deleted homozygously in 74% of the tumor cells

h) hepatic cells (in liver metastasis) in the same sarcomatoid MPM preparation S-12 as in panel g) showed normal DNA copy numbers

i) hematoxylin eosin stain of the sarcomatoid MPM liver metastasis (the case S-12), x10 magnification

j) IHC showed negative p16 staining for tumor cells as well as hepatic cells of the case S-12; stromal cells were not evaluated in sarcomatoid cases, x20 magnification
