# Supplementary material for: CDKN2A copy number and p16 expression in malignant pleural mesothelioma in relation to asbestos exposure
Source: BMC Cancer. 2019 May 28;19:507. doi: 10.1186/s12885-019-5652-y (PMC6537412; doi:10.1186/s12885-019-5652-y)
Supplement: Supplementary file 3 — A table - Relation of the CDKN2A copy number and p16 staining status in tumor cells of malignant pleural mesothelioma patients in general and with different asbestos-burden and in quality controls. (PDF 762 kb) [file 12885_2019_5652_MOESM3_ESM.pdf]

Additional File_3
"CDKN2A copy number and p16 expression in malignant pleural mesothelioma in relation to asbestos exposure"
by Kettunen E, Savukoski S, Salmenkivi K, Böhling T, Vanhala E, Kuosma E, Anttila S, Wolff H.
Eeva Kettunen, PhD; Finnish Institute of Occupational Health, Finland; Mar 19, 2019, eeva.kettunen@ttl.fi

Additional File 3. Relation of the *CDKN2A* copy number and p16 staining status in tumor cells of malignant pleural mesothelioma patients in general and with different asbestos-burden and controls.

		Malignant pleural mesothelioma							
		All cases[a]		High exposure study group[b]		Low exposure study group[c]		Quality controls[d]	
		***CDKN2A* copy number**							
		abnormal[e]	normal	abnormal[e]	normal	abnormal[e]	normal	abnormal[e]	normal
**p16**	negative, n (%)	42 (69)	2 (3)	27 (77)	1 (3)	10 (71)	0 (-)	2 (29)[f]	0 (-)
**staining**	positive, n (%)	10 (16)	7 (12)	6 (17)	1 (3)	0 (-)	4 (29)	2 (29)[g]	3 (42)[h]
κ[i]		0.428		0.146		1.000		0.461	

[a]All malignant mesothelioma for which we had both *CDKN2A* copy number and p16 staining result

[b]Patients' pulmonary asbestos fiber count ≥1.0 x10^6 fibres per gram dry lung (f/g)

[c]Patients' pulmonary asbestos fiber count 0-0.5 x10^6 f/g

[d]Quality controls had either serous ovarian or serous peritoneal carcinoma, adenocarcinoma of the lung or pleomorphic liposarcoma

[e]*CDKN2A* copy number was considered as abnormal if homozygous deletion was shown in >20% of the cells or hemizygosity in > 20% of the cells, or monosomy of chromosome 9 in ≥50%

[f]Adenocarcinoma of the lung and pleomorphic liposarcoma

[g]Serous ovarian and serous ovarian or peritoneal carcinoma

[h]Serous ovarian carcinoma, serous peritoneal carcinoma, and serous ovarian or peritoneal carcinoma

[i]Cohen's kappa (κ) coefficient.
