# Supplementary material for: CDKN2A copy number and p16 expression in malignant pleural mesothelioma in relation to asbestos exposure
Source: BMC Cancer. 2019 May 28;19:507. doi: 10.1186/s12885-019-5652-y (PMC6537412; doi:10.1186/s12885-019-5652-y)
Supplement: Supplementary file 4 — A pdf file of images of FISH and IHC preparations of malignant pleural mesothelioma (MPM). (PDF 2607 kb) [file 12885_2019_5652_MOESM4_ESM.pdf]

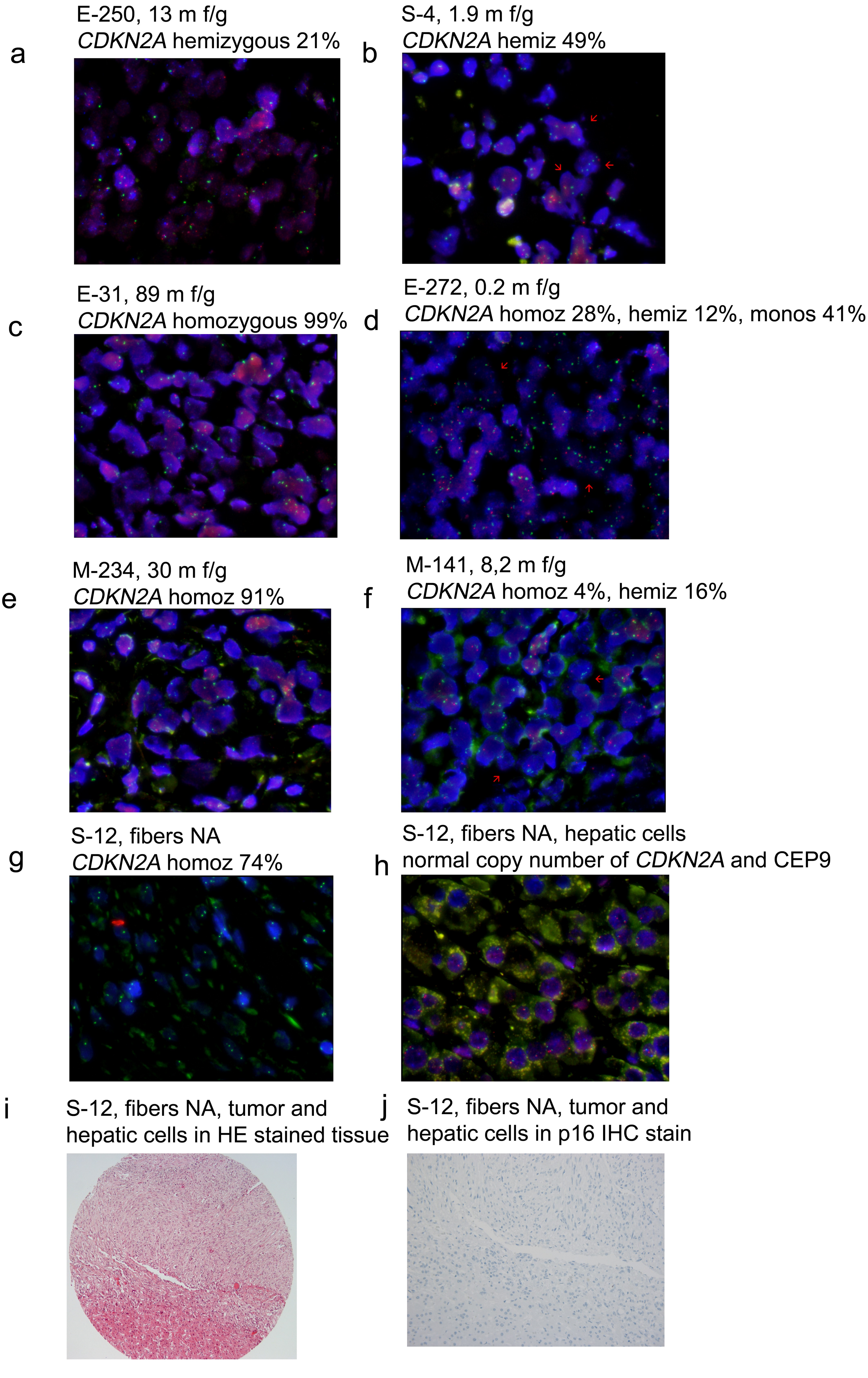
a
E-250, 13 m f/g
CDKN2A hemizygous 21%
b
S-4, 1.9 m f/g
CDKN2A hemiz 49%
c
E-31, 89 m f/g
CDKN2A homozygous 99%
d
E-272, 0.2 m f/g
CDKN2A homoz 28%, hemiz 12%, monos 41%
e
M-234, 30 m f/g
CDKN2A homoz 91%
f
M-141, 8,2 m f/g
CDKN2A homoz 4%, hemiz 16%
g
S-12, fibers NA
CDKN2A homoz 74%
h
S-12, fibers NA, hepatic cells
normal copy number of CDKN2A and CEP9
i
S-12, fibers NA, tumor and
hepatic cells in HE stained tissue
j
S-12, fibers NA, tumor and
hepatic cells in p16 IHC stain
